# Steroid Hormone Sensitivity in Reproductive Mood Disorders: On the Role of the GABA_A_ Receptor Complex and Stress During Hormonal Transitions

**DOI:** 10.3389/fmed.2020.479646

**Published:** 2021-01-18

**Authors:** Sophie Schweizer-Schubert, Jennifer L. Gordon, Tory A. Eisenlohr-Moul, Samantha Meltzer-Brody, Katja M. Schmalenberger, Radoslaw Slopien, Anna-Lena Zietlow, Ulrike Ehlert, Beate Ditzen

**Affiliations:** ^1^Center for Psychosocial Medicine, Institute of Medical Psychology, University Hospital Heidelberg, Heidelberg, Germany; ^2^Practice for Psychoendocrinology and Psychotherapy, Heilbronn, Germany; ^3^Department of Psychology, University of Regina, Regina, SK, Canada; ^4^Women's Mental Health Research Program, Department of Psychiatry, University of Illinois at Chicago, Chicago, IL, United States; ^5^Department of Psychiatry, University of North Carolina, Chapel Hill, NC, United States; ^6^Department of Gynecological Endocrinology, Poznan University of Medical Sciences, Poznan, Poland; ^7^Department of Psychology, University of Zurich, Zurich, Switzerland

**Keywords:** depression, stress, allopregnanolone, GABAR_A_ receptor, premenstrual, perinatal, perimenopausal, peripartal

## Abstract

Women worldwide are two to three times more likely to suffer from depression in their lifetime than are men. Female risk for depressive symptoms is particularly high during the reproductive years between menarche and menopause. The term “Reproductive Mood Disorders” refers to depressive disorders triggered by hormonal fluctuations during reproductive transitions including the perimenarchal phase, the pre-menstrual phase, pregnancy, the peripartum period and the perimenopausal transition.

Here we focus on reproductive mood disorders manifesting in adult life. We propose a research agenda that draws together several reproductive mood disorders and investigates which genetic, endocrinological, neural, and psychosocial factors can explain depressive symptoms during phases of hormonal transitions in women. Based on current research it is assumed that some women experience an increased sensitivity to not only fluctuations in reproductive steroids (estrogen and progesterone), but also stress-related steroids. We integrate both dynamics into the concept of “steroid hormone sensitivity,” expanding on the concept of “reproductive hormone sensitivity.” We suggest that a differential response of the stress steroid system including corticosteroids, neurosteroids, like allopregnanolone and the GABA-A Receptor complex, as well as a differential (epi)genetic risk in serotonergic and GABAergic signaling, are moderators or mediators between changes in the reproductive steroid system and the physiological, affective, and cognitive outcomes manifesting in reproductive mood disorders. We point to the lack of research on the role of psychosocial factors in increasing a woman's stress level and at some point also the sensitivity of her stress steroid system within the etiology of Reproductive Mood Disorders.

Drawing together the evidence on various reproductive mood disorders we seek to present a basis for the development of more effective pharmacological, social, and psychological treatment interventions and prevention strategies for women susceptible to these disorders. This could pave the way for new research as well as medical and psychological teaching and practice- such as a new type of Practice for Gynecological Psychoneuroendocrinology- with the aim of working on and ultimately offering more integrative forms of support not yet available to women suffering from depression during hormonal transitions. In medical history women have been left alone with this integrative challenge.

## Introduction

Women all over the world are two to three times more likely to suffer from depression in their lifetime compared to men (1). This risk is particularly high during the reproductive years between menarche and menopause. This suggests that mood disorders triggered by changes in reproductive steroid hormones during reproductive transitions- including the perimenarchal phase with first-onset depression (2–4), the menstrual cycle, pregnancy, the peripartum period and the menopause transition- may account for an important proportion of this increased risk. Indeed, an estimated 13–19% of reproductive-aged women experience clinically significant premenstrual mood disturbance each month (5, 6); 25% experience significant mood symptoms during or following pregnancy (7) and approximately 45–68% of women experience clinically significant mood symptoms during the menopausal transition (8). While each disorder is unique, it is also believed that increased sensitivity to fluctuations in reproductive steroid hormone levels represent an underlying etiologic process common to all three reproductive phases (9). For this reason, this cluster of mood disorders occurring in the context of reproductive transitions are frequently referred to as “Reproductive Mood Disorders” (RMDs) in academic circles and will be the focus of this review.

We will begin by briefly introducing the key neuroendocrine factors featured in all RMDs and will then proceed in the following sections with a more in depth look at three RMDs occurring in adult life: premenstrual dysphoric disorder (PMDD), peripartum depression (PPD), and perimenopausal depression (PMD). We will also describe reproductive steroid hormone environments that characterize each reproductive transition, and the evidence for the involvement of increased sensitivity to reproductive steroid hormones in the etiology of each disorder, as well as the role of stress-relevant mechanisms like the hypothalamic pituitary adrenal (HPA) axis, neurosteroids and GABAR_A_R receptors. Furthermore, we end each section with genetic considerations specific to each RMD as well as neuroimaging findings. As a final step, we seek to draw together the evidence from the various RMDs to outline a common etiology, while also discussing potential differences between them. Building on earlier initiatives to discuss a shared etiology between the RMDs [e.g., (10–12)], we expand the previously established concept of reproductive steroid hormone sensitivity toward the concept of “steroid hormone sensitivity,” in order to also integrate the contributions of stress-related steroid hormones and the neurosteroid allopregnanolone (ALLO) at the GABAR_A_R receptor, a central switch between the reproductive hormonal system and the HPA Axis. We argue that women suffering from RMDs not only have an abnormal reaction to normal reproductive steroid hormone changes, but also an abnormal reaction to normal stress steroid hormone mechanisms including ALLO signaling. Psychosocial factors will briefly be discussed as a pivotal compound in the role of stress sensitivity in these disorders. Taken together, our perspective on RMDs is conceptualized with the ultimate goal of informing their improved diagnosis and treatment as well as improving the early identification of women who are at the highest risk of RMDs to enable prevention.

## Key Neuroendocrine Factors In Reproductive Mood Disorders

Sex differentiation in the prevalence of mood disorders begins with puberty (13). Before adolescence, the rates of depression are similar in girls and boys or even slightly higher in boys (12). However, after menarche, a sharp rise in the prevalence of mood disorders and suicidal behaviors in women can be observed (14, 15). Although perimenarchal mood disorders could clearly be considered a RMD, only limited research is available on the underlying psychoneuroendocrinological mechanisms [see for instance Smith (16)]. However, as will be presented in the Neuroimaging Section The Role of Serotonergic Function in RMDs: Possible Direct and Interactive Pathways below, the findings from this strand of research clearly distinguish pubertal depression from the other adult RMDs. Therefore, we here focus on RMDs manifesting in adult life.

### Sensitivity to Changes in Reproductive Steroids

**Figure 1:**
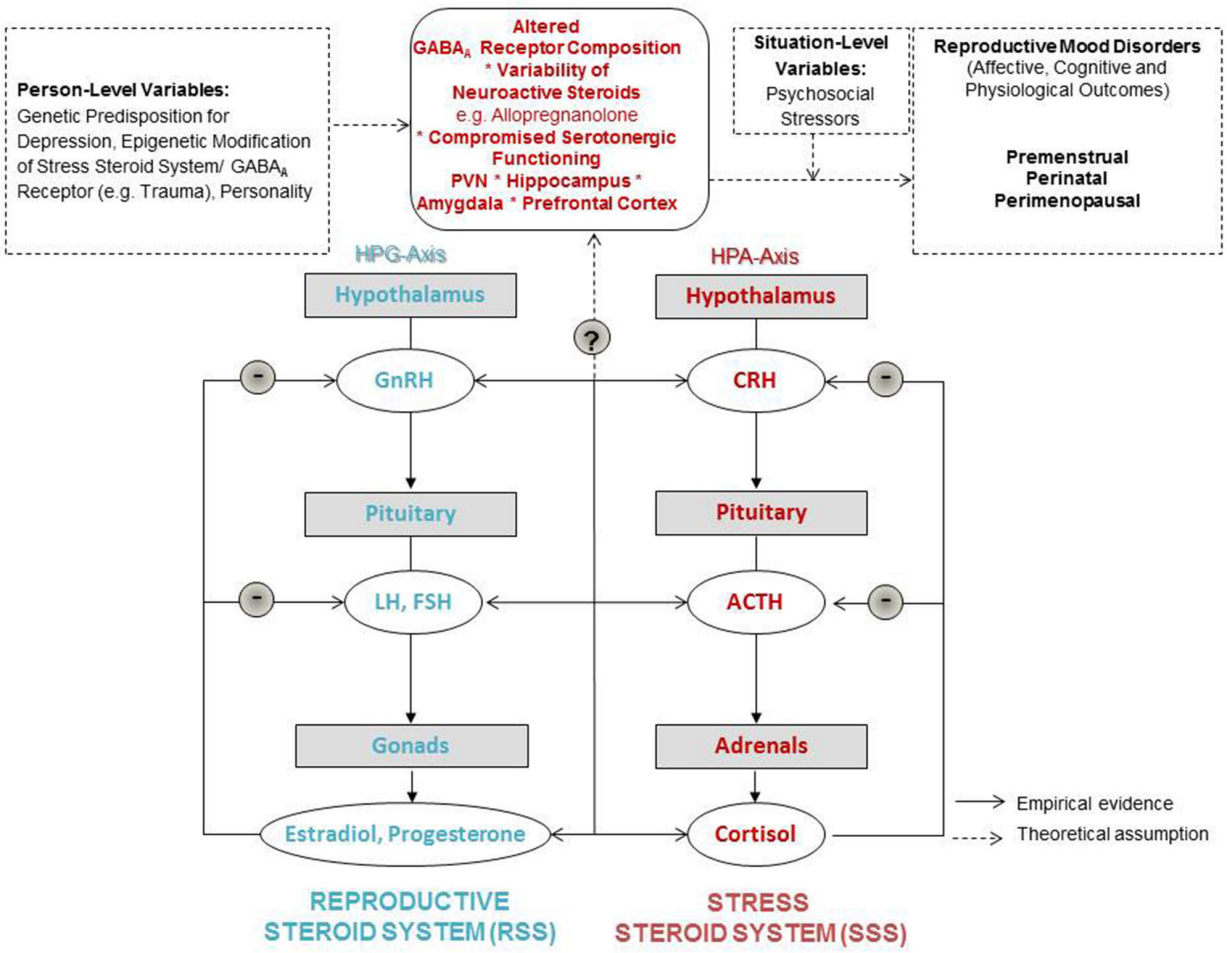
Neuroendocrine modulation of reproductive mood disorders.

Reproductive steroid hormones including estrogens, progesterone and androgens are regulated by the hypothalamic-pituitary-gonadal (HPG) axis. Briefly, as the initial part of the HPG axis, the hypothalamus releases gonadotropin-releasing hormone (GnRH), which stimulates the anterior pituitary to produce and secrete the gonadotropins- luteinizing hormone (LH) and follicle-stimulating hormone (FSH). LH passes the bloodstream and stimulates the release of reproductive steroid hormones from the gonads followed by a negative feedback loop, as illustrated in Figure 1. The HPG axis not only regulates reproductive function, but also other physiological functioning, and influences emotion and cognition [as e.g., reviewed in Gurvich et al. (17)].

The marked increase in mood symptoms during times of reproductive transitions suggests that common underlying reproductive steroid hormone-based mechanisms influence mood symptoms in women. The role of reproductive steroid hormones in regulating the switch into dysfunctional affective states in a susceptible group of women has been well-established by studies showing abnormal symptomatic responses to experimental steroid hormone manipulations in those with PMDD (18), peripartum depression (19), and perimenopausal depression (20), however, the source of this susceptibility remains unknown. After nearly two decades of research devoted to clarifying the etiology of RMDs, there is compelling evidence that these disorders are not characterized by absolute *levels* in reproductive steroid hormones but result from an increased sensitivity to *changes* in reproductive steroid hormones instead [see for instance (10, 11, 19–22)]. Therefore, research on RMDs moves more and more toward a focus on understanding affective responses to *changes* in reproductive steroid hormone levels and the underlying neuroendocrinological and genetic mechanisms underlying these responses (23). Also, the ratio between the reproductive steroid hormones estrogen (E2) and progesterone (P4) has been shown to be crucial in the development of psychiatric disorders (24).

### Sensitivity to Changes in Stress-Related Steroid Hormones, Neurosteroids, and the GABA_A_ Receptor Complex

Psychosocial stress is positively associated with the development and severity of mood disturbances during all phases of reproductive transition (25–29). In line with this, there is consistent research showing dysregulations of the hypothalamic-pituitary-adrenal (HPA) axis in depressive disorders. The effector hormones of the HPA axis (most prominently cortisol) act on glucocorticoid- and mineralocorticoid receptors in the central nervous system and provide *negative* feedback to further regulate HPA axis activation [(30); also see Figure 1]. Overall, a hyperexcitable HPA axis (for instance as a result of significant early life stress or trauma) has been found to be a common feature of depression (31, 32).

The γ-aminobutyric acid (GABA) system plays a critical role in inhibiting the HPA axis at the level of the paraventricular nucleus (PVN) of the hypothalamus, with GABA as the dominant inhibitory neurotransmitter (33–35). Neuroactive steroids (NAS) are metabolites of cholesterol or steroidal precursors that constitute potent and rapid allosteric modulators of the GABAR_A_ Rreceptor (36). Among them, a neuroactive P4 metabolite, the neurosteroid 3α-OH-5αβ-pregnan-20-one, or allopregnanolone (ALLO) is of particular interest in this context (37). ALLO acts as a positive allosteric modulator of the GABAR_A_ Rreceptor with potent anxiolytic and tranquilizing effects. Animal research demonstrates an HPA axis-dampening effect of ALLO: ALLO administration in rats attenuated stress-induced release of adrenocorticotropic hormone (ACTH) and cortisol; ALLO also attenuates the stimulated release of the corticotropin-releasing hormone (CRH) and prevents hypothalamic CRH gene expression in adrenalectomized rodents [reviewed in Girdler and Klatzkin (38)].

During the fetal and infant period, important changes in gene expression related to intracellular chloride concentration resulting in altered GABAergic tone have been observed. Change in gene expression affecting these mechanisms seems to occur for some women, but not all and it can be argued that this can influence a woman's risk for RMDs (37). During early development, GABA is depolarizing and mostly excitatory due to high [Cl^−^]. Here it plays a key role by regulating a number of processes including the migration, morphological maturation, and differentiation of neurons (39). GABA mediates Cl(-)dependent inhibitory postsynaptic potentials and alterations in these mechanisms e.g., due to epigenetic changes due to excessive stress as described below are related to GABA's potential implication in the pathogenesis of disorders, RMDs (37).

Apart from such alterations in GABA-activated CL- channels, another aspect that is relevant to a better understanding of a potential mechanism sensitizing some women for RMDs is that certain stem cells continue to be excitatory in adulthood. In relation to this aspect of stem cells it can be said that cognitive functions in adults are modulated by hippocampal neurogenesis which in adult humans occurs exclusively on the level of the dentate gyrus (40). The only product of hippocampal neurogenesis are granule cells as the principal excitatory neurons of the dentate gyrus (41). They provide excitatory input to the pyramidal cells of CA3 (42). The precursor cells from which adult neurogenesis originates receive synaptic input from various other brain regions including dopaminergic fibers from the ventral tegmental area, serotonergic projections from the raphe nuclei, acetylcholinergic input from the septum, and GABAergic connections from local interneurons (43, 44).

It has been argued that genetic or epigenetic modifications of the GABAR_A_ Rreceptor may contribute to a woman's sensitivity to ALLO and, in turn, her risk for mood disturbances in response to reproductive steroid hormone fluctuations. For instance, stress in childhood or puberty has been argued to modify the expression of the αR_4_Rβδ GABAR_A_ Rreceptor and plasticity on mood and cognition in the face of stress (45). This particular receptor subunit combination is sensitive for ALLO. Further, the biosynthesis of ALLO has been argued to be affected by excessive stress, which in turn compromises cognitive and affective functioning (46). As Locci and Pinna (46) emphasize, stress-induced down-regulation of ALLO biosynthesis and changes in the GABAR_A_ Rreceptor have been related to disorders like posttraumatic stress disorder (PTSD) and depression. On another note, lower serum ALLO levels were found to be associated with higher serotonergic binding in the prefrontal cortex regulating higher cognitive functions and top-down regulation of emotions, while higher ALLO levels were associated with lower alertness (47). Sundstrom Poromaa et al. suggest that this could explain the higher well-being in the follicular phase of the menstrual cycle. Further, sensitivity (and resulting affective vulnerability) might be related to epigenetic changes in genes determining serotonergic functioning (48). However, there is no scientific evidence yet, that abnormalities in serotonergic signaling in depressive responses (49) apply to women suffering from RMDs.

In sum, it is theoretically possible that increased mood sensitivity to ALLO fluctuation (caused by fluctuations in reproductive steroid hormones), manifesting as altered GABAergic tone, would have important consequences for mood outcomes in the face of stress. As such, dysregulation in the stress steroid system is considered in the current manuscript as a potential player in the RMDs, perhaps mediating the relationship between reproductive steroid hormone fluctuation as well as subsequent ALLO fluctuation and mood disturbance in a subset of women. Very recently, a comprehensive review on the mechanisms between GABAergic dysfunction and the HPA axis in the context of depression has been provided (37). However, as it is noted in this review, most of the presented studies have employed male subjects only, and the findings may not be translatable to the female sex. Given the sex differences in stress reactivity (50), behavior (51), and a potential differential in the GABAR_A_RRsR Rexpression linked to reproductive steroid hormones (52), our review focuses on findings from female subjects.

Concerning the role of ALLO in these mechanisms, at this point, it could be helpful to clearly separate out two issues: First, paradoxical effects of (normal levels of) ALLO due to a epigenetic modification of αR_4_Rβδ GABAR and, and second, compromised ability to synthesize ALLO (i.e., low ALLO levels) due to epigenetic modification concerning 3a-HSD and other enzymes needed to synthesize ALLO from P4. They are likely to represent two separate issues, since the first is about sensitivity of the system to normal ALLO changes and the latter is about deficits in levels of ALLO. As will be outlined below, the RMDs described in this manuscript differ in the availability of studies demonstrating the former vs. the latter. In sum, the role of the epigenetically modified GABAR_A_R receptor composition for the activation of the receptor by neurosteroids needs further elucidation in order to better understand RMDs.

### Interactions Between the HPA and the HPG Axes: The Central Role of GABAergic Mechanisms

A link between HPA axis activation and HPG axis suppression has long been established via a suppression of GnRH signaling to the pituitary and corresponding decrease in FSH and LH dampening gonadal activity (53). In depressed females (as well as males) HPA axis activity is inversely related to HPG axis activity (31). Based on this, a bi-directional relationship between the experience of stress and activation of the HPG axis has been suggested: Variability in sex steroids has been related to stress vulnerability and perimenstrual psychiatric risk (24, 54), impaired affective adjustment during the peripartum period (55, 56) and perimenopausal depressive symptoms (20).

At the level of the central nervous system, we consider GABAergic mechanisms as mediators between the HPA and HPG axes in order to advance our understanding of RMDs. The GABAergic Deficit Hypothesis of Major Depression posits that defects in GABAergic neural inhibition can contribute to the etiology of depressive symptoms and that future antidepressant therapies could be improved by focusing on the restoration of GABAergic neurotransmission (57, 58). For comprehensive reviews of the role of the GABAR_A_ RReceptor, the relevant subunits in mood disorders in the aftermath of stress as well as the role of ALLO and allotetrahydrodeoxycorticosterone (THDOC) in this please see the work of Maguire [e.g., (37)] as well as Locci and Pinna (46).

Both, the HPA and the HPG axes are regulated by GABAergic transmission at the level of CRH and gonadotropin-releasing hormone (GnRH) neurons. Higher neurosteroid sensitivity has been found in GABAR_A_ RRs incorporating the δ subunit. Therefore, stress-derived neurosteroids at GABAR_A_ Rreceptors containing δ subunits have been hypothesized to regulate the cross-talk between the HPA and the HPG (59). As suggested from the research above, fluctuations in P4, and subsequent fluctuations in ALLO could be involved in both HPA and the HPG axes functioning. However, evidence on the role of ALLO at central GABAR_A_R receptors, their interplay between the HPA and the HPG axes and the genesis of RMDs is very limited, because ALLO mechanisms cannot be measured in the living human brain. Based on the available data, it can be hypothesized that women susceptible to RMDs show higher sensitivity to stress during phases of reproductive steroid hormone variability. This sensitivity corresponds to altered ALLO-dependent functioning at the GABAR_A_R receptor in the PVN (see Figure 1). In fact, there is evidence of a shift in somatic GABA currents (ER_GABA_R) in parvocellular neurons in the PVN after chronic early life stress (60). In sum, chronic stress seems to lead to compromised GABAergic control of the HPA axis.

### Neuroimaging Findings of Reproductive Mood Disorders

Although much of the literature on the neurobiology of MDD, which did not make a distinction between men and women, may still provide valuable insight for RMDs, there are very few functional brain imaging in fact comparing dysfunctional neuronal networks in depressed men and women [e.g., (61)]. Pubertal depression that may manifest as first-onset depression in the perimenarchal phase was found to produce neuroimaging results that significantly differ from the adult Reproductive Mood disorders (PMDD, PPD, PMD) and are more akin to activation patterns observed in Major Depressive Disorder (MDD) (62). The identification of a female depression biotype, as well as differences between female mood disorders across the reproductive cycle can provide essential insights concerning their potential respective treatment. The sub-sections of this article that portray each of these disorders also include a section reviewing the neuroimaging findings for the specific reproductive mood disorder manifesting in adult life.

### The Role of Serotonergic Function in RMDs: Possible Direct and Interactive Pathways

Altered serotonergic function has also been observed among women with RMDs, and therefore these mechanisms should be considered when building theoretical and empirical models of RMD. Deficits in serotonergic function are observed across RMDs [as reviewed in Schiller et al. (63)], and pharmacologic fMRI studies have demonstrated that estrogen and progestin administration (vs. placebo) cause increased 5-HT2A binding in brain areas critically involved in emotion regulation, including the anterior cingulate cortex, dorsolateral prefrontal cortex, and lateral orbitofrontal cortex (64). The interactions between sex hormones and the serotonergic system in both healthy women and those with RMDs are extensively reviewed elsewhere [e.g., (63, 65)]. In later sections on various adult reproductive mood disorders, we will discuss both serotonergic abnormalities and potential interactions with the GABAergic system as they relate to specific RMDs.

It could also be the case that the serotonergic and GABAergic systems interact to cause RMD symptoms (66). As reviewed by Birzniece et al. (66), a clear interaction between the GABAergic and the serotonergic systems has been described in the hippocampus. In this particular area of the brain serotonin neurons have been found to frequently end at inhibitory GABAergic interneurons (67). In the context of the relationship between the GABAergic and the serotonergic system in reproductive mood disorders (discussed in more detail below) there have been findings that *in vivo* administration of low doses of a 5HT1A receptor agonist with anxiolytic effects enhances GABA stimulated ClP^−^ Puptake in cortico-hippocampal synaptoneurosomes (68). More experimental studies are needed to determine the relevance of these interactive mechanisms in the regulation of behavior and RMD symptoms. In this paper, we retain a primary focus on the GABA/ALLO system while acknowledging the importance of the serotonin system in RMDs, as there is a possibility that there are important interactions between the GABAergic and serotonin systems in the etiology of RMDs.

## Premenstrual Mood Disorders

Premenstrual mood disorders include premenstrual syndrome (PMS), PMDD, and premenstrual exacerbation (PME) of ongoing mood disorder (69). It is estimated that 13–19% of naturally cycling women experience clinically significant emotional PMS symptoms (5, 6) and that about 5% meet diagnostic criteria for PMDD (70), a severe form of PMS recently formalized as a new diagnosis in the DSM-5 (71). PMDD is characterized by the recurrent luteal phase emergence of clinically significant affective symptoms that become minimal or absent after the onset of menstruation (72–74). This “on-off” pattern in which symptoms are fully *confined* to the luteal phase has been emphasized as a critical diagnostic feature for selecting “pure” PMS/PMDD (i.e., without comorbidities) in research studies; however, there may be important variability in the exact timing of the onset and resolution of symptoms in PMDD and related syndromes (75). With respect to PME prevalence, prospective data from epidemiologic studies indicate that roughly 60% of women with depressive disorders demonstrate clinically significant PME of at least one depressive symptoms (76). Therefore, premenstrual mood disorders affect a large portion of women. While PMDD was not coded for in the ICD-10, PMDD is new in ICD-11 where it is put under gynecologic disorders, but is cross-listed as a depressive disorder. This might cause confusion, since there is no evidence for a gynecologic pathology in PMDD. ICD-11 will not come into effect until the year 2022, which means that it is currently not used in most places. At the moment there is an ICD-11 Browser made available by the World Health Organization (WHO), where these new categories can be found (World Health Organization, ICD-11 Browser, Version 2019).

### Sensitivity to Changes in Reproductive Steroids in PMS/PMDD

The reproductive steroid hormones E2 and P4 change in a predictable fashion across the prototypical 4-week menstrual cycle. In the first week, which starts with menstrual bleeding, both E2 and P4 are low and stable, followed by a large abrupt peak in E2 at the end of the second week, just prior to ovulation. In the third week, formation of the corpus luteum after ovulation leads to a two-week elevation of P4 and secondary elevation of E2. In the final week, both E2 and P4 plateau and then fall precipitously in the final few days prior to menses onset. In the paragraphs that follow, we outline what is known about the pathophysiology of PMS/PMDD. Since the pathophysiology of PME of depression has been found to be unique from PMDD but has yet to be clarified in its own right (i.e., PME of depression is resistant to various effective treatments for PMDD; Bixo et al. (77), Freeman et al. (78), Peters et al. (79)], we will focus our review below on the role of reproductive steroid hormones in PMS/PMDD (i.e., those with luteal phase confinement of symptoms).

As yet, studies have failed to demonstrate consistent abnormalities in E2, P4, or ALLO hormone levels among women with PMS/PMDD. Instead, premenstrual mood disorders are thought to be caused by an aberrant response to normal fluctuations in reproductive steroid hormones. A series of clinical trials demonstrate that GnRH agonists effectively treat the symptoms of PMDD by creating a medical menopause characterized by low, stable levels of reproductive steroid hormones [reviewed in Wyatt et al. (80)]. However, experimental studies demonstrate that addback of luteal phase levels of E2, P4, or their combination causes a resurgence of PMDD-like symptoms not observed in controls and not precipitated by placebo (18). Recently, Schmidt et al. (22) have also demonstrated that this symptomatic response to addback of reproductive steroid hormones is time-limited, remitting after 1 month of stable addback. This series of experiments suggests that it is the postovulatory *changes* in reproductive steroid hormones—and not the elevated luteal hormone levels themselves—that precipitate symptoms in PMS/PMDD.

Further, experimental work demonstrates that PMS/PMDD are caused by an abnormal sensitivity to normal postovulatory *surges* in reproductive steroid hormones rather than perimenstrual reproductive steroid hormone *withdrawal*. Schmidt et al. (81) tested the effects of reproductive steroid hormone withdrawal on symptoms of PMDD in three groups of women; one third received placebo in the midluteal phase, and the other two thirds received mifepristone, a competitive P4 receptor antagonist that causes menses (breakdown of endometrium) and luteolysis (involution of the corpus luteum and associated E2 and P4 withdrawal). Of those receiving mifepristone, half also received human chorionic gonadotropin (HcG), which rescued the corpus luteum, preventing reproductive steroid hormone withdrawal (while still allowing mifepristone-induced menses for blinding purposes); the other half of those on mifepristone received a placebo, which resulted in both mifepristone-induced menses and mifepristone-induced luteolysis with attendant early reproductive steroid hormone withdrawal. The authors reported no significant mood differences between the three groups (midluteal induction of early menses and hormone withdrawal with mifepristone + placebo injection, midluteal induction of early menses but normal midluteal hormone levels with mifepristone + HcG injection, and a natural midluteal phase without menses or hormone withdrawal on dual placebos). This indicates that neither initiation of menses nor induction of hormone (and attendant ALLO) withdrawal alone underlie symptoms of PMDD. In combination with the results of studies described above, we conclude that an abnormal post-ovulatory sensitivity to *surges* in reproductive steroid hormones (and not a perimenstrual sensitivity to reproductive steroid hormone withdrawal) likely precipitates the symptoms of PMS/PMDD.

### Sensitivity to Changes in ALLO and the HPA Axis in PMS/PMDD

Although general psychiatric populations have been found to show reduced biosynthesis of ALLO (46), a recent experimental metabolomics study demonstrated normal P4 metabolism to GABAergic neurosteroids (e.g., ALLO) in PMS/PMDD relative to controls (82). Instead of being caused by a general reduction in ALLO levels (e.g., similar to that observed in depression or PTSD), evidence is accumulating to support the notion that PMS/PMDD are caused by an abnormal neural sensitivity to normal ALLO changes. A recent randomized controlled trial demonstrated preliminary evidence for efficacy of dutasteride in PMDD, a 5α-reductase inhibitor that prevents the metabolism of P4 to its GABAergic neurosteroid metabolites such as ALLO (83). This indicates that it is an altered sensitivity to postovulatory ALLO surges, and not solely E2 or P4 surges, that triggers symptoms of PMS/PMDD.

This altered response to ALLO surges in PMS/PMDD might be caused by abnormal or insufficient plasticity of the GABAR_A_R receptor in response to the postovulatory rise in ALLO [e.g., an upregulation of the α4βδ GABAR_A_R receptor; Shen et al. (84)]. This argument is supported by preliminary evidence for the efficacy of UC1010 (Sepranolone), which acts as an antagonist at the neurosteroid binding site of the GABAR_A_R receptor at which ALLO is active, thereby preventing the adverse effects of ALLO surges in PMDD (77). Notably, these combined results would appear to rule out insufficient ALLO biosynthesis (either peripherally or in the CNS) as the cause of PMS/PMDD, since blockade of the neurosteroid binding site is therapeutic rather than further triggering PMS/PMDD symptoms. In sum, it appears that symptoms of PMS/PMDD are probably related to an abnormal regulation of the GABAR_A_R receptor in response to postovulatory surges in ALLO.

Perimenstrual studies demonstrate that GnRH-agonist suppression of reproductive steroid hormones blunts the normative HPA axis response to stress in healthy women, and that addback administration of luteal levels of P4—and not E2—recapitulate the luteal phase potentiation of the HPA axis response to stress (85, 86). Since ALLO is known to limit the extent and duration of the HPA axis response to stress, it is likely that luteal phase increases in P4 potentiate the HPA axis response through other (e.g., genomic) mechanisms (87).

While the HPA axis and related cortisol output has been extensively studied in observational studies of PMS/PMDD, there is no consistent evidence for abnormal cortisol levels or responses to stress in PMS/PMDD [reviewed in Kiesner and Granger (88)]. Rigorous experimental studies (in which women with and without PMDD are tested during ovarian suppression with GnRH agonist, E2 addback, and P4 addback) demonstrate no differences in reproductive steroid hormone regulation of the HPA axis among women with PMS/PMDD compared to controls, including normal effects of administration of reproductive steroid hormones on output of CRH by the hypothalamus, ACTH by the pituitary, and glucocorticoids by the adrenals, as well as normal effects of administration of reproductive steroid hormones on glucocorticoid receptor feedback (85). Therefore, this may be an area of pathophysiology in which PMS/PMDD differs from other mood disorders such as unipolar depression and may also differ from the pathophysiology of peripartum or perimenopausal mood episodes (compare e.g., also sections HPA Axis Dysregulation and the Neurosteroid Hypothesis of Peripartum Depression and HPA Axis and GABA-ergic Mechanisms in Perimenopausal Depression).

Despite normal regulation of the HPA axis by reproductive steroid hormones (85), there is some evidence to indicate that trauma and recent life stressors increase the severity of hormone-related symptom expression in PMS/PMDD. Cross-sectional studies have observed a correlation between traumatic experiences and retrospectively-self-reported PMDD symptoms [a method known to have a very high false positive rate; Pilver et al. (89)]; however more rigorous studies in which PMDD was prospectively-diagnosed did not find increased exposure to trauma in individuals with PMS/PMDD (90). PMDD patients with high ALLO levels have been found to show blunted nocturnal cortisol levels in comparison to healthy controls who had low ALLO serum concentrations. It also has been argued that diurnal secretion of cortisol may be influenced by ALLO levels during the luteal phase (91). However, these findings do not replicate in another experimental study on PMDD, where affected women did not show altered ALLO metabolism following ovarian suppression and E2 or P4 addback (82). Nonetheless, a longitudinal study of patients with prospectively-diagnosed PMDD found that the strength of the daily link between P4 levels and symptoms was stronger in patients with histories of trauma, which may indicate that trauma increases the *severity* (rather than the occurrence) of hormone sensitive mood symptoms in PMS/PMDD (92). In addition to historical trauma exposure, current life stressors may exacerbate or prolong the mood effects of hormone sensitivity in women with PMS/PMDD. In one observational study, cycles preceded by higher-than-usual perceived stress showed greater premenstrual increases in mood symptoms (93). Another prospective study of medical students who were (or were not) beginning a stressful night shift assignment found greater increases in premenstrual mood *changes* among students starting the stressful rotation (94). Since the HPA axis has been found to be normal in PMS/PMDD, more work is needed to understand how internal and environmental stressors may interact with hormone sensitivity to increase cyclical mood symptoms.

### Brain Imaging Findings on PMDD

Neuroimaging studies of PMDD remain rare and of mixed quality [for a thorough review and critique of this literature, see Comasco and Sundström-Poromaa (95)]. However, a few patterns of interest have emerged and have begun to shed light on some possible neurobiological underpinnings of PMDD. A few studies comparing gray matter volumes between women with PMDD and healthy controls have observed larger posterior cerebellum and increased gray matter density in the hippocampal cortex, as well as lower gray matter density in the parahippocampal cortex (96, 97). Some studies have noted differences in brain function between those with and without PMDD regardless of cycle phase, including altered activation in the dorsolateral prefrontal cortex (98) and increased amygdala response to negative stimuli (99). Other studies show differences in how the cycle impacts brain function in PMDD. In a proton magnetic resonance spectroscopy study comparing PMDD and controls, cortical GABA decreased from the follicular to the luteal phases in controls, but increased from the follicular to the luteal phase in PMDD (100). Amygdala function may also respond abnormally to progesterone in PMDD; one study found that GABAergic progesterone metabolites predicted lower amygdala reactivity to social negative pictures in healthy controls, whereas the opposite relationship was found in those with PMDD (99). Finally, one study found that, relative to healthy controls, women with PMDD showed greater late luteal phase increases in activation of the left insula during a cognitive processing task (101). In sum, while there are some promising findings that may point to the underlying neurobiology of hormone sensitivity in PMDD, more systematic, and experimental imaging work is required to move this area forward.

### Interactions Between the Serotonergic and the GABAergic system in PMDD

As discussed by Birzniece et al. (66) SSRIs may increase inhibitory processes in the limbic structures of the brain involved in the emotional as well as cognitive regulation by hyperpolarization of neuronal membranes enhancing GABA-stimulated Cl^−^ uptake. Women diagnosed with PMDD have been shown to have a decreased sensitivity toward GABA_A_ receptor active substances, especially during the luteal phase (102), when altered serotonergic activity is also observed [reviewed in Hantsoo and Epperson (103)]. Serotonin reuptake inhibitors represent an effective treatment, especially by means of intermittent administration in the luteal phase (104). One possible mechanism is that the SSRI treatment increases metabolism of progesterone to allopregnanolone and normalizes the tolerance to neurosteroids observed during the luteal phase in PMDD (105). However, there is also some evidence that the benefit of SSRIs in PMDD is dependent on their serotonergic mechanisms (106), since coadministration of the serotonin receptor antagonist metergoline (vs. SSRI alone) is able to undermine the benefit of SSRIs in PMDD. Therefore, more work is needed to determine whether alterations of serotonin and GABA systems represent additive or interactive risk factors for PMDD.

### Genetic Factors in PMS/PMDD

Although a few studies have identified single-nucleotide polymorphisms (SNP) that differentiate PMS/PMDD cases from controls (107, 108), caution should be exercised when interpreting such studies (109). However, with a focus on epigenetic alterations a recent study of lymphoblastoid cell line cultures from women with PMS/PMDD and controls has demonstrated notable abnormalities in the cellular epigenetic processing of reproductive steroid hormones in PMDD, including altered mRNA expression of several ESC/E(Z) complex genes, both in control samples and in samples treated with E2 and P4 (110). Epigenetic changes across the menstrual cycle and in response to hormonal manipulations may represent a critical area of research to move forward our understanding of the pathophysiology of PMS/PMDD.

## Peripartum Mood Disorders

Diagnostic codes for peripartum mood disorders in the ICD and DSM diagnostic manuals differ and there is not a consistent definition. The ICD-10 (111) restricts to either pregnancy or postpartum onset, while in the DSM-5 (71), there is a “peripartum onset” specifier that includes both pregnancy and postpartum in the affective disorders section. ICD-11 distinguishes between mild, moderate, and severe episodes of depression and also between single and recurrent episodes, as well as with and without psychotic symptoms that are supplemented with “current episode perinatal” in the codes ICD 11 6A70-6A71 (World Health Organization ICD-11 Browser, Version 2019).

Up to 25% of women experience significant depressive symptoms following pregnancy (7, 112), however, it is estimated that about 80% of postpartum depression (PPD) cases are not recognized and officially diagnosed, which means that only 20% of affected women receive the treatment they need. Notably, affective symptoms are often already present during pregnancy and the presence of such symptoms in pregnancy has been shown to be among the strongest predictors of PPD (113).

### Reproductive Hormonal Changes During the Peripartum Period

For a comprehensive overview concerning normal reproductive steroid hormone fluctuations see Schock et al. (114). It has long been hypothesized that withdrawal of reproductive steroid hormones occurring in the postpartum period plays an important role in the etiology of PPD. One of the first studies providing strong evidence of this used a hormone manipulation paradigm simulating the reproductive steroid hormone withdrawal after parturition among women with a history of PPD and among women without. It was shown that the same hormone manipulation procedure produced a significant increase in depressive symptoms among the women with a history of PPD but not among women without. This suggests that PPD may result from an increased sensitivity to the changes in reproductive steroid hormones that characterize the postpartum period (19). Also, there are several small RCTs finding hormone therapy to be an effective treatment for postpartum depressive symptoms which also supports the role of hormonal withdrawal in the etiology of PPD (115).

While the mechanisms by which withdrawal from E2 and P4 triggers depressive mood in a subset of postpartum women are not fully understood, there is a growing body of research suggesting that increased sensitivity to psychosocial stress may play a role. For example, a history of stressful life events is a strong predictor of the development of PPD (116). Furthermore, psychological (e.g., adjustment to the role as a mother) and physiological stressors (e.g., sleep deprivation) markedly affect subjective well-being, affective symptoms, and physiological stress responses after birth (117). In addition, standard laboratory experiments have shown that stress-susceptibility prior to birth can predict postpartum mood symptoms (118).

### HPA Axis Dysregulation and the Neurosteroid Hypothesis of Peripartum Depression

Stress increasing factors for depression and anxiety for instance during pregnancy have been reviewed as risk factors for these disorders, e.g., lack of partner or of social support, a history of abuse or of domestic violence; personal history of mental illness; unplanned or unwanted pregnancy; adverse events in life and high perceived stress; present and past pregnancy complications and pregnancy loss (119). In light of the important role that psychosocial stressors and increased sensitivity to such stressors likely play in the development of peripartum depression, it is not surprising that there is more and more evidence implicating alterations in HPA axis functioning in the etiology of peripartum depression (119). The cortisol response of pregnant women (and new mothers, respectively), with a vulnerability for depression during reproductive transitions may vary as a function of the social support they receive, as well as other stress reducing interventions they may have access to (120). Pregnant women suffering from depression show a significantly higher cortisol response to stressors compared to their controls (118). Pregnant women with prepartum depression and/or anxiety disorder measured in the third trimester have also been found to show a higher cortisol response to stress, but only in the case of comorbidity with e.g., anxiety disorders (121). Also, higher mid-term CRH levels were associated with PPD (122). Further, a significant correlation between the cortisol awakening response and major depression was found in pregnant women (123). Setting pre-partum and postpartum mood problems into context in peripartum research can contribute to our understanding of RMDs, as we see a continued rise in E2 in the third trimester accompanied by an increase in mood symptoms and ultimately also the peak in depressive symptoms in the second and third postpartum week where accumulated pre-partum psychiatric burden adds up with postpartum hormone withdrawal (124).

In recent years, research on peripartum depression has evolved toward examining the role of neurosteroids (e.g., ALLO and DHEA) in the genesis of the disorder and a “hormone-sensitive” phenotype of postpartum depression has been proposed [e.g., (125)]. Dehydroepiandrosterone (DHEA) plays a particular role in affective dysregulation and abnormal DHEA secretion has been found in major depression [e.g., (126)]. Also, DHEA has been found to have anti-depressive effects on both, men and women (127). However, the neurosteroid ALLO has increasingly moved into the center of attention. Regardless of PPD or a healthy state, postpartum women show reduced cortical GABA and ALLO, comparable to healthy women in their follicular phase (128), which also suggests that normal absolute hormone levels are not the underlying cause, but the fluctuation of hormones. There appears to be a vulnerability or sensitivity in a subpopulation of women to the development of peripartum depression (125). It has been shown that the increasing levels of neuroactive steroids (NAS) during pregnancy are crucially related to modifications in the expression of specific GABAR_A_ Rreceptor subunits (129). During pregnancy and after parturition, due to major changes in reproductive hormones, the expression of the GABAR_A_R receptor δ subunit is altered (52, 130, 131). ALLO levels increase during pregnancy and the GABAR_A_ Rreceptor δ subunit is downregulated. After parturition ALLO levels decrease rapidly, the GABAR_A_ R receptor δ subunit is recovered. Based on research with rodents it has been hypothesized that i0n women at risk for PPD, this regulatory mechanism seems to be compromised. For a comprehensive review on these mechanisms, also with respect to the stress axis we recommend the most recent work by Walton and Maguire (132). Altered NAS and GABA profiles have been found in women at risk for peripartum depression. Peripartum GABA levels in women at risk for PPD manifested at a significantly lower level compared to healthy controls (133). In pregnant women whose stress regulatory mechanisms are compromised with respect to this GABAR_A_R receptor complex (for genetic or epigenetic reasons), there can be extremely high stress levels. So, the vulnerability to stress-related psychological disorders like anxiety and depression increases significantly, depending also on the potency of exogenous stressors such as social stressors (119). Neurosteroids play a key role in endogenous stress modulation (134). In sum, as it is hypothesized that a flexible plasticity of GABAR_A_R receptor subunits is compromised in PPD women, suggesting a dysfunction in adapting to peripartal hormonal changes. This would also correspond with the key notion proposed by Rubinow and Schmidt (23) that RMDs represent a problem of switching between affective states.

### Brain Imaging Findings on Peripartum Depression

Women with PPD have been found to show altered functional connectivity and activity in brain areas key for executive functions as well as emotion and reward processing. For a comprehensive review of structural and functional connectivity and molecular imaging research please see Duan et al. (135). Neuroimaging findings on PPD center around a decreased resting state functional connectivity between the anterior cingulate cortex, amygdala, hippocampus, and the dorsolateral prefrontal cortex against the background of falling progesterone and ALLO levels after giving birth (136). First fMRI and spectroscopy studies have started to bring GABA and ALLO mechanisms on screen in peripartum depressed women (137). GABA levels were found to be correlated with marked differences in the connectivity of the dorsomedial prefrontal cortex within the default mode network, which also correlated with depression cores in PPD women (137). Also, compromised connectivity has been reported between the amygdala and prefrontal cortex, which implies dysfunctions in emotion processing (138). Concerning an overlap between MDD and PPD functional abnormalities, findings are conflicting. While some authors argue that the network dysfunctions are identical between the disorders (138, 139), others argue that only perimenarchal depressive disorders share the same neuroimaging findings with MDD as mentioned earlier (62). Here, it has to be considered that the majority of neuroimaging studies had included cases suffering from PPD and MDD as well. Future research will have to make clear distinctions in this respect. In particular the hypothalamus, the amygdala, the anterior cingulate, the orbitofrontal and dorsolateral prefrontal cortices, the insula and the striatum seem to be key areas of interest to the etiology of PPD (140). In sum, fMRI studies demonstrate hypoactivation of brain regions studied in women with postpartum depression compared to those without PPD (62).

### Interactions Between the Serotonergic and the GABAergic system in Peripartum Depression

The link between the serotonergic system and the GABAergic system in the peripartum period is a research area of increasing interest. Antidepressant treatment with SSRI's has been shown to restore plasma and CSF ALLO levels in association with improvements in depressive symptoms (141, 142). Therefore, increasing levels of ALLO may play an important role in the antidepressant and anti-anxiety pharmacological effects of SSRIs (46). In addition, the recent FDA approval of brexanolone (Zulresso) for the treatment of postpartum depression provides further evidence that a positive allosteric modulator of GABA-A improves depression in the postpartum period (143, 144). However, the relation between brexanolone treatment in women with PPD and associated serotonin levels has not yet been shown.

### Genetic Factors in Peripartum Depression

There is a growing interest in understanding the genetic signature of peripartum depression. The literature consists largely of smaller genetic epidemiological and linkage studies (145–149). More recently, a study of the heritability of PPD using the Swedish national twin register estimated the heritability of PPD to be 54 and 44% using twin and sibling designs, respectively. In this same population, the heritability of depression occurring outside of the peripartum period was estimated to be 32% (150). These findings suggest that one-third of the genetic contribution to peripartum depression was unique and did not overlap with the genetic component of depression outside of the peripartum period. Therefore, the increased heritability, as well as increased genetic overlap with other mood disorders, makes peripartum depression an interesting candidate for future genetic investigations. Genetic aspects represent an important person-level variable in a potential etiological model of reproductive disorders, as will be shown in the graphical abstract introduced below as a precursor to such an etiological model (section Discussion: From “Reproductive Steroid Hormone Sensitivity” to “Steroid Hormone Sensitivity”).

Underlying pathophysiology can be used to probe the heterogeneity of peripartum depression. We hypothesize there may be different phenotypes of peripartum depression. Evidence from the international PACT Consortium (Postpartum Depression: Action Toward Causes and Treatment), has worked to help define the heterogeneity of peripartum depression and has described different phenotypes based on severity of symptoms, timing of onset of depressive symptoms, and presence of suicidal thoughts, race, and ethnicity among others (151–153). The PACT Consortium is also interested in the underlying genetic signature of PPD and is currently working on a large-scale Genome-wide association study of PPD (154). These approaches will increase our understanding of phenotypic differences but there remains an important gap between defining phenotypes and understanding the underlying mechanistic differences.

Consequently, the next step is to investigate the underlying pathophysiology that may be unique to the observed heterogeneity in PPD. For example, it could also be hypothesized that women with a susceptibility to fluctuations in reproductive steroid hormones who already show signs of depression during pregnancy may not develop a natural tolerance toward ALLO changes during pregnancy and postpartum, which could imply that they show an increased sensitivity in their key receptors for ALLO. The recent approval of brexanolone, a formulation of ALLO, in the US for PPD is highly relevant to this hypothesis. The women studied in the clinical trials had severe PPD with onset of symptoms in the third trimester or within the first month postpartum (143, 144).

## Perimenopausal Mood Disorders

The menopausal transition describes the reproductive phase transitioning from regular menstrual cycles to the complete cessation of menses, which marks the onset of menopause. Between ages 42 and 55, nearly all women experience the menopause transition, which, on average, extends 5–6 years leading up to the last menstrual period [e.g., Avis and McKinlay (155), Oldenhave et al. (156), Treloar (157)]. A recent review article identified 12 cross-sectional studies comparing rates of elevated depressive symptoms in pre- and peri-menopausal women and concluded that 45–68% of perimenopausal women, vs. only 28–31% of premenopausal women, report clinically significant elevations in depressive symptoms (8). Currently, perimenopausal depression does not have a diagnostic code that is distinct from major depression in either the ICD-10 (111) or the DSM-5 (71). ICD-11 still does not code for perimenopausal depression- this RMD would have to be coded by “Other specified menopausal and perimenopausal disorders, GA20Y” (World Health Organization, ICD-11 Browser, Version 2019).While the Center for Epidemiologic Studies Depression Scale (CES-D) is the questionnaire that is most commonly used to assess depressive symptoms in the menopause transition, there has been a call for a menopause-specific scale that would better take both physical and affective symptoms into consideration (158). In this review, we focus on reproductive hormonal fluctuations as a critical factor in perimenopausal problems. However, for the sake of completion- and as will also be referred to in the concluding research outlook- other stress responsive systems also need to be taken into consideration. For instance, the immune system, as well as the thyroid hormonal system can be at the root of climacteric symptoms (159). So it is of utmost importance to rule out other causes like thyroid disorder or autoimmune disorder (160) in women presenting with perimenopausal depression. Also, the links between the reproductive hormonal and the immune system have to be kept in mind, in particular in the context of Hormone Therapy in the Perimenopausal Transition (161). Fluctuations in Progesterone levels have also been shown to alter immune responses and susceptibility to infections at diverse mucosal sites including the genital, gastrointestinal, and respiratory tracts. So the immunomodulatory effects of Progesterone-based compounds need to be given thorough consideration in the treatment of perimenopausal symptoms (162). Reproductive hormonal changes seem to be at the core of those interactions with other hormonal system disorders as well as immunological disturbances, so for this review, we focus on fluctuations in reproductive hormones.

### Reproductive Steroid Hormone Changes of the Menopause Transition

The menopausal transition is characterized by several hormonal changes, triggered by a diminishing number of ovarian follicles and fluctuating levels of FSH (163). Beginning in the early menopause transition and continuing into the late transition, is the appearance of menstrual cycles that are characterized by elevated luteal phase E2 levels, which can reach levels that are as high as double those generally seen in the late follicular phase (164, 165). E2 levels in the early follicular phase, on the other hand, have been shown, at times, to reach lower levels than typically observed in reproductive-aged women (166). Furthermore, the low-E2 early follicular phase lengthens due to a delayed ovarian response to FSH, resulting in a longer cycle (167). While P4 levels remain intact throughout the early menopause transition, luteal P4 is lower, on average, throughout the late menopause transition (168). Anovulatory cycles, characterized by low P4 but variable E2 levels, also become increasingly common, with 60–70% of cycles being anovulatory in the late transition (169).

### Evidence that an Abnormal Sensitivity to Normal Perimenopausal Reproductive Hormonal Changes Contributes to Perimenopausal Depression

Several studies implicate extreme fluctuation in E2 in the development of perimenopausal depression. Perhaps the strongest evidence comes from a placebo-controlled study by Schmidt et al. (20), which experimentally induced E2 withdrawal using an E2 patch and observed a marked increase in depressive symptoms in the following weeks among women with a history of perimenopausal depression that had been responsive to E2, but not among those without. These findings are in line with three studies observing a relationship between greater E2 fluctuation and elevated risk for depressive symptoms (170–172), as well as a recent placebo-controlled RCT examining the efficacy of transdermal E2 in the prevention of depressive symptoms among perimenopausal and early postmenopausal women (173). In this 12-month study, the rate of clinically significant depressive symptoms was found to be lower among women assigned to transdermal E2 (100 ug/day) vs. placebo (17 vs. 32%). Interestingly, women in the early menopause transition were found to experience the greatest mood benefit, perhaps suggesting that its effects were due to its ability to stabilize the hormonal environment rather than increase low E2 levels.

### HPA Axis and GABA-ergic Mechanisms in Perimenopausal Depression

In light of the importance of both hormonal and psychosocial contributions to the development of perimenopausal depression, it has been hypothesized that the hormonal environment of the menopause transition, particularly increased E2 fluctuation, may interact with the stress axis to confer an increased sensitivity to psychosocial stress and increased vulnerability to mood disturbance (163). However, when compared to PMDD and peripartum depression, research directly examining this potential interaction is greatly lacking. In one study cited above (163), a significant interaction was found between E2 fluctuation and the presence of major stressful life events such that E2 fluctuation was predictive of depressive mood among women experiencing one or more events but not among those without baseline stress. Furthermore, the 12-month RCT of transdermal E2 cited above found that the mood benefits of E2 were enhanced among women with greater baseline stressful life events (173). While two studies to date suggest that basal cortisol levels are unrelated to depressive mood in the menopause transition (126, 174), there is some evidence that altered diurnal cortisol patterns may be associated with perimenopausal depressive symptoms. For example, an ancillary of the Study of Women Across the Nation (SWAN) including 408 midlife women, found that depressive symptom score was associated with a flatter diurnal cortisol slope (175). A second study has also observed a relationship between greater weekly changes in E2 and elevated morning cortisol among women with current perimenopausal depression but not among euthymic controls (171, 172). This latter finding would be consistent with the notion that increased HPA axis activation following E2 (and, in turn, ALLO) fluctuations may play a role in perimenopausal depression.

The potential involvement of the GABAergic system and GABAergic neurosteroids, such as ALLO, in the etiology of perimenopausal depression remains largely theoretical as little clinical research directly testing its contribution has been conducted. In a rat model, ovariectomy has been shown to greatly reduce brain ALLO concentrations while administration of 17β estradiol (0.1 or 1 ug per day for 14 days) has been shown to restore ALLO to pre-ovariectomy levels in the hippocampus and hypothalamus (176), an effect that is likely explained by E2's modulation of the enzymes involved in the conversion of P4 to ALLO, 5α-reductase, and 3α-hydroxysteroid dehydrogenase (177). It is therefore possible that the shift between the more extreme E2 levels that characterize the menopause transition, either alone or in conjunction with P4, may result in a more dramatic change in ALLO concentrations than would be seen in a typical menstrual cycle, thus triggering depressive mood in a subset of women for whom the GABAR_A_R receptor fails to adjust to the rapid change in neurosteroid levels. These women may also be more prone to depressive mood when first experiencing a hypogonadal state in the late perimenopausal and early postmenopausal phases: in line with this possibility, a recent study examined the correlation between serum ALLO levels and mood in 140 late perimenopausal and early postmenopausal women and found that ALLO was negatively correlated with feelings of guilt among the early postmenopausal women (29).

### Brain Imaging Studies in Perimenopausal Depression

To date, there has been little research examining the neural correlates of perimenopausal depression. One study by Berent-Spillson et al. (178) compared brain activation patterns in euthymic pre-, peri-, and postmenopausal women in response to an emotion identification task. Perimenopausal women were found to have a negative bias in identifying the emotions exhibited by the neutral faces; furthermore, both peri- and postmenopausal women exhibited less limbic activation and greater activation of the tempo-parietal-occipital junction (178), a pattern considered to indicate a more cognitively-mediated decision-making process than an emotionally-mediated one. The cognitively-mediated decision-making process was associated with greater depressive symptoms across all three groups. Laboratory sessions occurred in the early follicular (low E2) phase among cycling women, though E2 levels did not correlate with any of the outcomes assessed, suggesting that these differences are not related to differences in hormone *levels*, but that perhaps greater hormonal instability and/ or hormonal withdrawal is the hormonal driver behind these observed differences. Other imaging studies, while not conducted in perimenopausal women *per se*, have used experimental hormonal manipulations in other populations to inform our understanding of the menopause transition. These studies have primarily focused on the involvement of the serotonergic system and are therefore described in the next section.

### Interactions Between the Serotonergic and the GABAergic system in Perimenopausal Depression

As with PMDD and PPD, the potential involvement of other neurotransmitter systems apart from GABA have been investigated in perimenopausal depression, with the serotonergic system receiving the most attention. Neocortical serotonin transporter binding (resulting in greater serotonin reuptake and therefore lower serotoninergic tone in this region) has been found to increase in response to an experimentally induced hypogonadal state mimicking that observed in the late menopause transition (49). Furthermore, cortical serotonin transporter binding has been found to decrease (179) while 5-HTR_2A_R receptor binding has been found to increase (180) following the administration of estrogen therapy in postmenopausal women. One final study examining serotoninergic influences in perimenopausal depression examined the interaction between tryptophan depletion and estradiol treatment on brain activation during an emotion identification task among early postmenopausal women (181). Specifically, it was found that tryptophan depletion reduced activation of the dorsolateral prefrontal cortex and medial frontal/cingulate gyrus compared to sham depletion but that this effect was eliminated with the administration of estradiol. Also relevant to the potential involvement of the serotonergic system in perimenopausal depression is one study comparing the volume of monoamine oxidase A, an enzyme involved in the degradation of serotonin, in the prefrontal cortex of pre-, peri-, and postmenopausal women (182). This study found that on average, women in the perimenopausal age range (41–51 years) had 34% greater monoamine oxidase A volume compared to young reproductive-aged women and 16% greater than older women.

The above studies implicating the serotonergic system in the etiology of perimenopausal depression are consistent with research finding that selective serotonin reuptake inhibitors (SSRIs) are an effective treatment for perimenopausal depression [see Maki et al. (183) for review]. Taken together, these research findings suggest that the serotonergic system has a role to play in the etiology of perimenopausal depression; however, in light of the complex interactions that are well-documented between the GABAergic and serotonergic system, this certainly does not preclude the simultaneous involvement of the GABAergic system as more thoroughly provided above. Interactions between the two systems remain to be explored in depth in the perimenopausal context.

### Genetic Factors in Perimenopausal Depression

Current research examining potential genetic contributions to the development of perimenopausal depression is limited. The largest study to date involved 1,538 pre- and perimenopausal women ages 42–55 participating in the Study of Women's Health Across the Nation (SWAN). In this study, specific polymorphisms of three genes involved in the metabolism of E2 and estrone (E1)–the CYP1A1 gene among Caucasian and African American women, the 17HSD gene among Chinese women, and the CYP 19 gene among Japanese women – were found to be associated with an increased risk of elevated depressive symptoms (184). However, neither the ESR1 nor the ESR2 gene, respectively, encoding estrogen receptors α and β, were found to be relevant for risk of depressive symptoms. A second study including 488 women ages 42–68, of which 156 were perimenopausal (54 depressed and 102 controls), observed depression-related differences in the frequency of polymorphisms for the MAO-A and MTHFR genes, which are relevant for monoamine oxidation and methylation, respectively. However, several other genes, including the ESR1 gene, several genes involved in serotonergic transmission (5HTR2A, 5HTR1B, and 5HTR2C), and the GABRB1 gene, which codes for a subunit of the GABAR_A_R receptor, were not found to be relevant for depression in the perimenopausal subgroup (185). However, statistical power to detect such effects may have been limited. A third study including 391 Chinese women (191 with major depressive disorder, 200 controls) found that, among women ages 40–60, a gene-by-environment interaction between negative life events and allelic variations of the ESR2 gene could be seen in relation to major depressive disorder. However, no such interaction was observed among younger women, raising the possibility that the hormonal environment of the menopause transition, when combined with negative life events, may trigger depressive mood in women with a genetic predisposition involving the ESR2 gene. However, there is clearly much work to be done in clarifying the role that genetics play in the etiology of perimenopausal depression. Future research that more carefully defines the menopause transition via menstrual bleeding patterns using the Stages of Reproductive Aging Workshop +10 (STRAW+10) criteria (186), excluding postmenopausal women, is needed. The STRAW+10 guidelines define the early menopause transition based on the appearance of a menstrual cycle length 7+ days shorter or longer than usual and the late menopause transition based on the occurrence of two skipped cycles, but <1 year since the last menstrual period.

## Discussion: From “Reproductive Steroid Hormone Sensitivity” to “Steroid Hormone Sensitivity”

Based on the research findings presented in this review, the following conclusions can be made with a fair degree of confidence. First, rigorous hormonal manipulation studies have demonstrated strong evidence that increased mood sensitivity to changes in reproductive steroid hormones plays a key role in the etiology of all three RMDs discussed—PMS/PMDD (18), PPD (19), and PMD (20). The evidence that increased sensitivity to ALLO fluctuation mediates the link between reproductive steroid hormone changes and mood disturbance is quite strong in the context of PMDD (83) and PPD (143). However, further work is needed to examine the role that sensitivity to ALLO fluctuation triggered by E2 or P4 fluctuation may play in the development of perimenopausal depression. Although it seems likely that genetic and epigenetic contributions play an important role in conferring an increased sensitivity to ALLO fluctuation, we are far from having a clear and consistent picture about the specific genes involved.

A second assertion that can be made at this stage of research on RMDs is that proximal psychosocial stress increases susceptibility for mood disturbance in the context of reproductive transitions. In the case of PPD and perimenopausal depression, there is some evidence that HPA axis dysregulation may either be a correlate of depressive symptoms or may play a partial role in the development of mood disturbance. However, the evidence for its role as a primary mechanism underlying the etiology of these RMDs is relatively weak. More work is needed, in particular also on PMDD, to identify the mechanisms by which psychosocial stress may increase risk for and/or exacerbate RMDs. Overall, the GABAR_A_R receptor in specific subunit combinations has been found to be a clear center piece in these mechanisms between the stress hormonal axis and the reproductive hormonal axis (37).

Third, the serotonergic system has also been implicated in all three RMDs. However, we do not consider this body of research as being contradictory to the simultaneous involvement of GABAergic neurosteroids. Indeed, there is considerable evidence suggesting that the serotonergic system is directly modulated by these neurosteroids, including ALLO (46, 66). In sum, clear linkages between the GABAergic and the serotonergic systems have been found in the hippocampus, where serotonin neurons frequently end at inhibitory GABAergic interneurons (67). Also *in vivo* administration of low doses of a 5HT1A receptor agonist that comes with anxiolytic effects enhances GABA stimulated ClP^−^ Puptake in cortico-hippocampal synaptoneurosomes (68).

Recent literature presented earlier in this manuscript and also in the following closes the circle to chronic stress or trauma in childhood and youth, that has been established as a clear risk factor for RMDs. Life stress that causes developmental insults alters exactly those GABA-stimulated chloride mechanisms in CRH neurons that constitute a key mechanism in the GABAergic control of the HPA axis. These insights link to and build forth on another research tradition that might benefit research on RMDs: research on the psychobiology and molecular genetics of resilience (187). The stress resilience of women with RMDs seems to be severely compromised, which relates to the very few existent studies on chronic stress and early life stress reported earlier in this article in this context. In a recent and comprehensive review on this topic, Hodes and Epperson (188) elaborate on the existing findings concerning the difference between men and women in response to significant life stress. The findings presented in this review confirm that life stress leading to developmental insults, in particular during childhood and puberty, is unmasked in women during hormonal fluctuations in reproductive transitions. In women, this then manifests in stress-related disorders such as depression, anxiety and posttraumatic stress disorder. In contrast, prenatal and early postnatal stress in men tends to manifest in other symptoms, such as those pertaining to the autism spectrum disorders as well-attention-deficit/ hyperactivity disorder. Further research in this particular context of reproductive transitions and life stress in women could hold valuable keys for improving the mental health of women affected by RMDs.

Also, the brain imaging evidence presented in this article shows that there is far more research needed to get a clear picture of shared activation patterns in RMDs, as the studies for each RMD are to heterogenous. In sum, a couple of aspects have to be kept in mind when drafting a common etiological model for RMDs: a clear difference in brain activation patterns has been found between adult RMDs and premenarchal mood problems, in that the latter was more akin to MDD. So it seems sensible to draft a common etiological model for adult RMDs only. In brain imaging studies GABA also played a central role, in that GABAergic fluctuations were clearly tied to fluctuations in the reproductive hormonal system. Also, decreasing ALLO levels were found to be linked to changes in brain activation pattern. So, brain imaging research also warrants the inclusion of the GABAergic system and its activating neurosteroids into an etiological model of RMDs. Not only the GABAergic mechanism at the level of the PVN, activation patterns in the hippocampus, but also compromised connectivity between the amygdala and the prefrontal cortex played a major role in RMDs. Some researchers found an identical network dysfunction and blunted brain activation patterns for all RMDs. Concerning serotonergic functioning hypogonadal states were found to come with greater serotonergic uptake and decreased serotonin transporter binding blunting serotonergic functioning in brain imaging studies. Both, the serotonergic as well as the GABAergic system need to find a place in a joint etiological model.

In light of the above, in Figure 1 we present a graphical abstract that could serve as a stepping stone for etiological models for RMDs. In this graphic we integrate the supposed shared mediators and moderators of RMDs. The model expands on reproductive steroid hormone sensitivity as a concept suggesting that rather than absolute steroid hormone levels, it is the sensitivity toward relative changes in reproductive steroid hormones (indicated by plus and minus symbols) which drives affective, cognitive, and physiological outcomes (11). These outcomes can also be expected to be closely related and depend on situation-level variables of women such as psychosocial stressors, as well as person-level variables suches the genetic predisposition and epigenetic modifications of the systems introduced and labeled in the following.

Therefore, we wish to add a focus on a sensitivity to stress via altered GABAR_A_ Rreceptor and ALLO functioning on a central nervous system level. Thus, we find it helpful to propose to expand on the concept of reproductive steroid hormone sensitivity toward introducing a more integrative concept of “steroid hormone sensitivity”: We include both, a sensitivity in the reproductive steroid system (RSS) as well as what we label “the stress steroid system” (SSS). The dynamics between those two steroid systems needs in-depth investigation. There are to date no integrative data on these neuroendocrine dynamics and their influence on women's everyday life. Shared sensitivity to normal reproductive steroid and neurosteroid concentrations seems the focal point in the Stress steroid system for all RMDs. Therefore, we position GABAR_A_ Rreceptor composition as the central switch between the RSS and the SSS in our graphical abstract, as this receptor regulates both systems as reviewed earlier in this manuscript. The composition and plasticity of this receptor seems key in whether the cross-talk between the two steroid systems is flexible and adaptable during hormonal fluctuations. Whether a woman with a vulnerable genetic or epigenetic set-up including compromised GABAergic modulation develops RMDs then depends on the social context, including its stressors and supportive factors.

## Conclusion

For all three RMDs manifesting in adult women's life—PMDD, PPD and perimenopausal depression—there is strong evidence that biological factors (e.g., hormonal, genetic) and psychosocial factors (e.g., daily-life burden/stressors) interact to predict depressive symptoms. However, so far, much of the research has tended to examine these mood disorders from one perspective or the other, failing to consider how an individual's biological vulnerability may interact with her social environment to predict the risk for depression. As a result, clinicians frequently have a limited view of RMDs: obstetricians/gynecologists may tend to view these disorders as being purely hormonal, responsive only to pharmacological intervention, while counselors and social workers may fail to appreciate the degree to which biological influences play a role.

Furthermore, despite increasing evidence that the RMDs may have much in common, they are still being investigated in isolation, with research teams specializing in one disorder or the other. This is also reflected in the diagnostic manuals ICD [e.g., (111)] and DSM-5 (71), where these disorders are spread across different diagnostic categories of codes. RMDs still have to find their adequate place in diagnostic manuals and it is continued research on RMDs that is paving the way for this.

Expanding our perspective toward the more comprehensive concept of “Steroid Hormone Sensitivity” that integrates interactions between both, the reproductive steroid system (RSS) and the stress steroid system (SSS) might serve as a neuroendocrine basis for a better understanding of the underlying differential stress sensitivity experienced by affected women in daily life. Psychosocial stress effects manifesting in affective as well as cognitive symptoms may be of particular importance in these mechanisms. Compromised in their cognitive functioning and in turn stressed by these disabling symptoms, these women may spiral down in a vicious circle between affective, physiological and cognitive symptoms ultimately resulting in what we diagnose as RMDs. Researchers in both research traditions on depresssion- those on reproductive steroids as well as those on stress steroids- seem to be more and more turning toward abnormalities in receptor plasticity. The GABAR_A_ RReceptor could be the key switch between the reproductive and the stress steroid system with mounting evidence at a subunit level within this receptor complex: a compromised plasticity required during hormonal fluctuations. Concluding from our review, the role of the δ subunit within the αR_4_Rβδ GABA Receptor seems to be key in a successful endocrine modulation of reproductive transitions in an environment marked by social stressors.

Working with a common etiological model may not only help us all to proceed in our joint understanding of RMDs, but it may also facilitate the development of new therapeutic approaches combining psychosocial and neuroendocrine aspects. Detecting psychosocial stressors such as the lack of support and finding solutions for them is one of those aspects. From a neuroendocrine point of view, elevating ALLO levels has been discussed as a therapeutic approach to stress-related disorders (46). The FDA approval of a formulation of ALLO (Brexanolone) in the treatment of postpartum depression has, therefore, broad implications for our field. Others have also focused on the translocator protein (18kDa) (TSPO) which is key for neurosteroidogenesis (189) or a novel, synthetic, neuroactive steroid SGE-516 to improve postpartal depression-like symptoms in mice (190). Overall, the role of GABAR_A_ RReceptor composition for the activation of the receptor by neurosteroids (37, 46) should be at the center of pharmacological endeavors. In this context there is still far more in-depth evidence needed for the epigenetic regulation of GABAergic transmission. In depression research addressing GABAergic deficits means moving from the mere treatment of the symptoms of depression toward correcting causal neurochemical imbalances.

From a methodological point of view, we encourage the use of parallel methodologies across the three RMDs and suggest testing for common neuroendocrinological, genetic and psychosocial contributors to these disorders. Several methodological challenges will have to be carefully taken into account: the adequate measurement points in all disorders; interaction effects between reproductive steroid hormones and sex hormone binding globulin (SHBG), dehydroepiandrosterone DHEA and androgen measurement; inflammation markers; the choice of biomarkers for genetic phenotyping (such as serotonin, genotyping receptors, but also transporters); other neurotransmitters, targeting GABA receptors and ALLO in humans. With respect to psychosocial factors, confounding factors such as aging and self-image in the case of menopause or anxiety in younger women having to deal with family and workload have to be considered too. The growing high-risk demographic of single working mothers would be a good example, as the prevalence of depression in this group is significantly higher than in married controls due to chronically high stress levels (191). Sub-clusters of patients within the respective RMDs will have to be carefully distinguished as may have become clear in the course of this review. Current research that is exploring the crosstalk between Glucocorticoids, Reproductive Hormones and Immunity (192) may also benefit from picking up this thread of research on RMDs.

Ultimately, our review is meant to further establish RMDs as a phase-sensitive distinct clinical entity warranting the development of a new diagnostic category on RMDs in future editions of ICD and DSM. In particular, in the face of the compelling evidence available on perimenopausal depression, it is urgently necessary to insert a respective diagnostic category, as even the upcoming ICD-11 does not make any reference to this particular RMD. Integrating our knowledge on these health issues of women in a bigger picture allows us to take a psychoneuroendocrinologically informed stance on fundamental societal questions that have long been of pressing relevance. These include the differential social support and stress profiles in the context of different depression prevalence between men and women (120) and involves factors like the increase in stress-related diseases world-wide.

As we tried to show in this review, the hormonal as well as the psychosocial environment of the menopause transition is clearly implicated in the development of for instance perimenopausal depression. There is mounting evidence that the presence of psychosocial stress may interact with this hormonal environment to confer an increased mood vulnerability. Taking such a psychoneuroendocrinologically informed stance also calls for new teaching contents in medical and psychological faculties as well as a new type of practices for Gynecological Psychoneuroendocrinology (193). In this new type of practice treatment options will be offered that integrate neuroendocrinological knowledge with psychiatric, gynecological, urological, and social evidence as well as the latest insights of sexual medicine. This new type of practice supports women in finding adequate solutions to their problems, an extraordinarily demanding integrative effort of various medical, social and psychological services that women were left alone with for most of this world's medical history. In research, teaching and practice that takes such a multidisciplinary stance, a whole array of aspects can be taken into account in finding health solutions for women. For instance, also the aspects of pain and inflammation that are linked to depression (194) has never been elucidated in its link to altered GABAergic mechanisms found in RMDs. Neuro-orthopedic, gynecological as well as urological inflammations such as cystitis often represent masked depressive processes. These are aspects which would greatly benefit progress on RMDs in research and practice if addressed in such a multidisciplinary way. On the basis of a longitudinal, multi-disciplinary perspective on RMDs that takes psychosocial and neuroendocrine factors into account, far more effective prevention and intervention strategies including pharmacological, psychotherapeutic, and psychosocial approaches may be developed, with the aim to improve mental health of the many women in this world affected by reproductive mood disorders.

## Author Contributions

SS-S and BD: conception and design of the work. SS-S, JG, TE-M, SM-B, KS, RS, A-LZ, UE, and BD: drafting the article. SS-S, BD, and UE: critical revision of the article. SS-S, JG, TE-M, SM-B, KS, RS, A-LZ, UE, and BD: final approval of the version to be published. All authors contributed to the article and approved the submitted version.

## Conflict of Interest

The authors declare that the research was conducted in the absence of any commercial or financial relationships that could be construed as a potential conflict of interest.
